# The Coordination Abilities of New Cyclic Analogs of Somatostatin

**DOI:** 10.1007/s10989-016-9546-4

**Published:** 2016-07-23

**Authors:** Aleksandra Marciniak, Marek Cebrat, Justyna Brasuń

**Affiliations:** 1 Department of Inorganic Chemistry, Wroclaw Medical University, Borowska 211A, 50-556 Wrocław, Poland; 2 Faculty of Chemistry, University of Wroclaw, F. Joliot-Curie 14, 50-383 Wrocław, Poland

**Keywords:** Somatostatin, Somatostatin analogs, Copper complexes, Potentiometric measurements, Spectroscopy

## Abstract

Two new somatostatin analogs with a characteristic part of the sequence -c(Cys-Phe-Trp-Lys-Thr-Cys)- and with two histidine and two aspartic acid moieties in their structures were synthesized and analyzed in terms of their coordination abilities with copper (II) ions. Both peptides bind Cu(II) effectively. Ligands form 4N complexes with $\left\{ {{\text{N}}_{\text{Im}} ,{ 3} {\text{N}}_{\text{amide}}^{ - } } \right\}$ binding mode in a basic range of pH. But in spite of very similar sequences of the two peptides a significant difference in the effectiveness of the binding of copper (II) ions was observed.

## Introduction

Many previous studies show that since the discovery of somatostatin and its analogues interest in these peptides is still unabated. They are used in a new way of cancer treatment: peptide receptor radionuclide therapy (PRRT) which is a very promising method used to treat patients with neuroendocrine tumors. In PRRT, somatostatin analog is connected with radionuclides by using a chelator like DOTA or TETA and a linker (Teunissen et al. 2005).

**Fig. 1:**
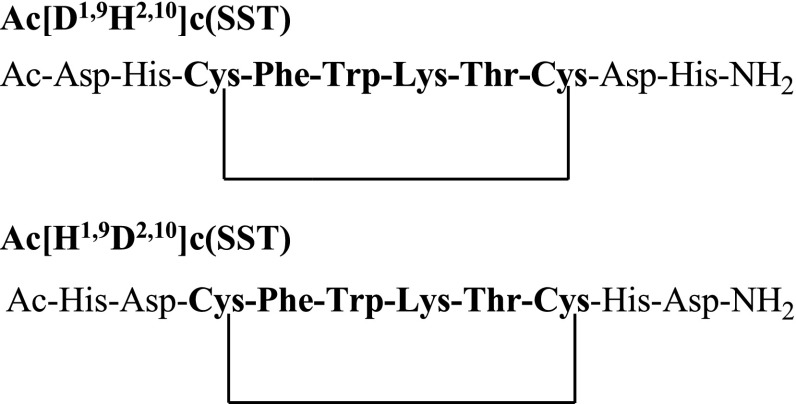
Sequences of analyzed peptides

**Fig. 2:**
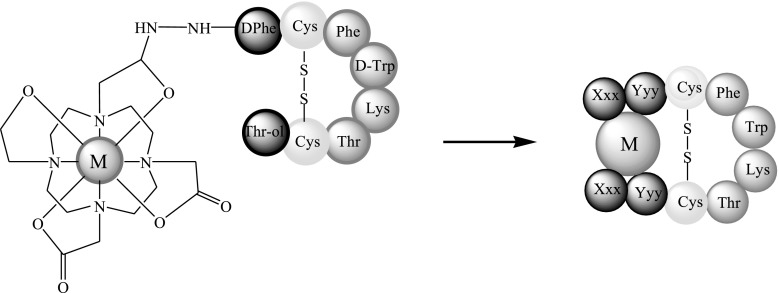
The difference in ways of complexes forming between somatostatin analogs used in medicine (*left*) and somatostatin analogs described in this study (*right*)

The subjects of the presented study are two somatostatin analogs with the part of the sequence: -c(Cys-Phe-Trp-Lys-Thr-Cys)- which is characteristic for the peptide hormone. Moreover, analyzed peptides have two histidine and two aspartic acid moieties in the peptide chains (Fig. 1). Four amino acids occurring between two cysteinyl moieties are responsible for biological activities and interactions with somatostatin receptors in this group of compounds (Pawlikowski and Melen-Mucha 2004). Furthermore, the presence of His and Asp amino acid residues, which are effective donors for metal ions, especially for copper (II) (Sovago et al. 2006, 2012) allows to create a binding site for a metal ion in the peptide structure. This way of the metal ion binding would allow to eliminate a linker and a chelator from precursors of radiopharmaceuticals potentially useful in PRRT (Fig. 2).

Our previous studies of linear somatostatin analogs show that analyzed ligands bind copper (II) ions effectively. But, unfortunately, cyclic complexes that were formed had structures not similar to natural hormone peptides with disulfide bridge between two cysteinyl moieties or the cyclic complex did not dominate in the physiological range of pH (Marchewka et al. 2012; Marciniak et al. 2012, 2014). Therefore, the analysis of cyclic ligands is the next step in searching of new somatostatin analogs.

## Materials and Methods

### Synthesis of the Peptides

Peptides were synthesized by the standard manual Fmoc solid-phase peptide synthesis method on the Fmoc-Rink Amide MBHA resin (0.65 mM/g, Iris Biotech GmbH). Synthesis was carried out in single-use plastic reactors (Intavis GmBH). Functional groups in the side chains of the amino acids used for the synthesis were protected as follows: Asp(OtBu), Cys(Acm), His(Trt), Lys(Boc), Thr(tBu), Trp(Boc). Subsequent Fmoc-protected amino acids (3 eq) were attached by using 3 eq PyBOP (1*H*-benzotriazol-1-yloxy)(tri-1-pyrrolidinyl)phosphonium hexafluorophosphate as a coupling reagent in the presence of *N*-hydroxybenzotriazole (3 eq) and diisopropylethylamine (6 eq) for 2 h at room temperature. Fmoc protecting groups were removed by 25 % piperidine in dimethylformamide. Acetylation of the N-terminal amino group was performed on the resin by 1:1 mixture of acetic anhydride and 0.4 M *N*-methylmorpholine in dimethylformamide. Final cleavage of the peptides was achieved by “Reagent K” (81.5 % trifluoroacetic acid, 5 % phenol, 5 % thioanisole, 5 % water, 2.5 % ethanedithiol, 1 % triisopropylsilane) in 2 h at room temperature. Crude peptides, with Cys residues still protected by Acm groups, were precipitated by cold diethylether, washed with ether, dissolved in water and lyophilized.

In order to remove Acm protecting groups from the side chains of Cys residues and to form a disulfide bridge, peptides were dissolved in 80 % acetic acid (2 mL of acetic acid for 2 mg of a peptide) and 20 eq of I_2_ dissolved in a small volume of 80 % acetic acid was added. The reaction was performed under nitrogen and its progress was monitored by ESI mass spectrometry until no more unoxidized peptide was observed. Mixture was diluted with the same amount of water and excess of I_2_ was extracted using CCl_4_. Water fraction containing peptide was lyophilized.

Peptides were purified by semipreparative HPLC using Varian ProStar apparatus equipped with TOSOH Bioscience C18 column (300 Å, 21.5 mm, 10 μm beads) and 220 nm UV detector. Water–acetonitrile gradients containing 0.1 % TFA at a flow rate of 7 ml/min. were used for the purifications. Final purity of the lyophilized peptides was >95 % by analytical HPLC (Thermo Separation Product; column: Vydac Protein RP C18, 250 Å, 4.6 mm, 5 μm; linear gradient 0–100 % B in 60 min., solvent A—0.1 % TFA in water, solvent B—0.1 % TFA in 80 % acetonitrile:water solution, UV detection at 220 nm). Chemical identity of the ligands was confirmed by ESI–MS on a Bruker micrOTOF-Q or Bruker apex ultra mass spectrometer. Analytical data of the synthesized peptides are given in Table 1.

**Table 1:** Analytical data of the peptides

Peptide	[M + H]^+^		[M + 2H]^2+^		[M + 3H]^3+^		R_t_^c^	Preparative gradient^d^
	Calc.^a^	Found^b^	Calc.^a^	Found^b^	Calc.^a^	Found^b^	(min)	
Ac[D^1,9^H^2,10^]c(SST)	1330.5092	n.o.	665.7582	665.7834	444.1746	444.1881	21.9	20–30 % B in 45 min
Ac[H^1,9^D^2,10^]c(SST)	1330.5092	n.o.	665.7582	665.7838	444.1746	444.1882	21.8	25–32 % B in 45 min

*n.o.* not observed

^a^Monoisotopic mas calculated for the indicated ion formed by the peptide

^b^Monoisotopic mas found by ESI–MS

^c^Retention time of the crude peptide found by analytical HPLC

^d^HPLC gradient used for the semipreparative purification of the peptide

### Potentiometric Measurements

Potentiometric measurements were carried out using Molspin pH-meter system with Mettler Toledo InLab 422 semimicro combined electrode at 25 °C calibrated in hydrogen ion concentration using HCl (Irving et al. 1967). The ligands concentration was 8 × 10^−4^ mol/L and pH-metric titrations were performed in 0.1 mol/L KCl solution using sample volumes of 1.2 mL. Alkali (NaOH) was added by using a 0.25 ml micrometer syringe. The concentration of NaOH was 0.1 mol/L. Stability constants and stoichiometry of the complexes were calculated from titration curves using the SUPERQUAD program (Gans et al. 1985). The pH range where precipitation was observed was omitted during the calculations. Due to this fact some stability constants were only estimated, which is indicated in the “Results” section.

### Spectroscopic Measurements

Visible spectra of complexes were recorded at 25 °C on Varian Carry 50 Bio spectrophotometer. The electron paramagnetic resonance (EPR) spectra were recorded on Bruker ELEXSYS E500 CW-EPR, X-Band spectrometer, equipped with ER 036TM NMR Teslameter and E 41 FC frequency counter. The EPR simulated spectra and all EPR parameters were obtained by the Biomolecular EPR Spectroscopy Software of Wilfred R. Hagen (Hagen 2009). Circular dichroism (CD) spectra were recorded on Jasco J-1500 magnetic circular dichroism spectrometer in 230–800 nm range. The same concentrations were used for both spectroscopic and potentiometric studies.

### Fluorescence Measurements

Cary Eclipse fluorescence spectrophotometer was used for fluorescence measurements with excitations at 280 nm for both peptides. The concentrations for peptides solutions were: 6.6 × 10^−5^ mol/L for Ac[D^1,9^H^2,10^]c(SST) and 6.7 × 10^−5^ mol/L for Ac[H^1,9^D^2,10^]c(SST). Measurements were made at 25 °C. The titration was performed at pH 8.0 and 11.0 as a function of metal concentration. The measurements were done for pure ligands and for solutions with ligand to metal ratios in the range of 1:0.1–1:4.0.

## Results and Discussion

Both studied peptides have five protonation constants assigned to two Asp, two His and one Lys amino acid residues (Tables 2, 4) and they are comparable to those found in the literature (Holm et al. 1996).

**Table 2:** Potentiometric, UV–Vis, and CD data for the Cu(II)/Ac[D^1,9^H^2,10^]c(SST) system at 25 °C, I = 0.1 mol/L (KCl)

Ac[D^1,9^H^2,10^]c(SST)	log *β*	log *K*	UV–Vis		CD	
			λ (nm)	ε (M^−1^ cm^−1^)	λ (nm)	Δε (M^−1^ cm^−1^]
HL	9.70 ± 0.02					
H_2_L	16.61 ± 0.03					
H_3_L	22.74 ± 0.03					
H_4_L	26.74 ± 0.04					
H_5_L	29.83 ± 0.04					
log*K*_Lys_		9.70				
log*K*_His_		6.91				
log*K*_His_		6.13				
log*K*_Asp_		4.00				
log*K*_Asp_		3.09				
CuH_2_L	20.86 ± 0.06		–	–	–	–
CuHL	~15.73 ± 0.03		–	–	–	–
CuL	~8.78 ± 0.09		–	–	–	–
CuH_−1_L	1.60 ± 0.06		582	59	626^a^	0.48
					487^a^	−0.26
					323^c^	0.45
					252^b^	5.94
CuH_−2_L	−7.05 ± 0.06		523	68	626^a^	0.88
			579	sh	491^a^	−1.11
					321^c^	0.97
					257^b^	6.29
CuH_−3_L	−17.24 ± 0.06		523	74	626^a^	0.88
			579	sh	491^a^	−1.11
					321^c^	0.97
					257^b^	6.29
CuH_−4_L	−29.24 ± 0.07		–	–	–	–
log*K*_CuH2L–CuHL_		5.13				
log*K*_CuHL–CuL_		6.95				
log*K*_CuL–CuH–1L_		7.18				
log*K*_CuH–1L–CuH–2L_		8.65				
log*K*_CuH–2L–CuH–3L_		10.19				
log*K*_CuH–3L–CuH–4L_		12.00				

The ligand concentration was 8 × 10^−4^ mol/L; 1:1 ligand to metal ratio

*sh* shoulder

^a^d–d transition

^b^N_Im_ → Cu(II) CT

^c^N^−^ → Cu(II) CT

**Fig. 3:**
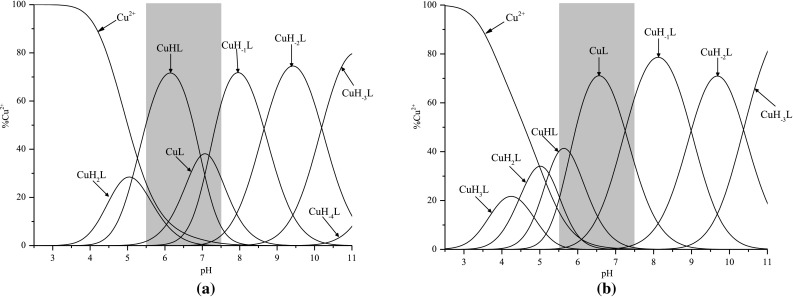
Species distributions curves for **a** Cu(II)-Ac[D^1,9^H^2,10^]c(SST) and **b** Cu(II)-Ac[H^1,9^D^2,10^]c(SST) at 25 °C; *I* = 0.1 mol/L KCl; ligand concentration 8 × 10^−4^ mol/L; ligands to metal ratio 1:1; area of precipitation is shown in *gray*

Analyzed somatostatin analog Ac[D^1,9^H^2,10^]c(SST) starts copper (II) ion binding around pH 4 and creates seven complexes (Fig. 3a). In the first one, CuH_2_L metal ion is probably coordinated by one nitrogen atom deriving from His residue. EPR parameters [A_||_ = 147 (cm^−1^) and g_||_ = 2.34, Table 3] confirm the existence of 1N complex. Moreover, the logβ* = 4.25 (where logβ* = log*β*_*CuH2L*_ − log*β*_*H2L*_) supports additional involvement of the carboxylate group from the Asp side chain. This value is different than logβ* for complexes where metal ion is coordinated only by imidazole nitrogen (Kapinos et al. 1998; Marciniak et al. 2014). Next form, CuHL, dominates in the system between pH 5.5 and 6 (Fig. 3a). The value of logβ*_CuHL–*HL*_ = 6.03 may be connected with the involvement of the second imidazole donor in copper (II) binding (Brasuń et al. 2007). In this conditions CuL complex also exists in the system. The value of log*β* = 8.78 (Table 2) may support involvement of the first amide nitrogen donor from the peptide chain (Fig. 3a). Unfortunately, above pH 5 precipitation was observed in the solution and it continued to pH 7.5. Molecules have a neutral charge there and this could be the reason of the precipitation (Hashempour et al. 2010). Therefore, determination of spectroscopic parameters for these two forms was not possible.

**Table 3:** The EPR parameters obtained by simulation of spectra in Cu(II)/Ac[D^1,9^H^2,10^]c(SST) system

Species parameters	CuH_2_L {N_Im_}	CuH_−1_L {N_Im_, 2N^−^}	CuH_−2_L {N_Im_, 3N^−^}	CuH_−3_L {N_Im_, 3N^−^}
g_z_ (g_||_)	2.340	2.225	2.194	2.193
g_x_, g_y_ (g_**⊥**_)	2.079	2.057	2.042	2.042
A_z_^Cu^ (A_||_)^a^	147	187	199	199
${\text{A}}_{\text{x}}^{\text{Cu}} ,{\text{ A}}_{\text{y}}^{\text{Cu}} ({\text{A}}_{ \bot } )^{{^{\text{a}} }}$	8	22	23	23

^a^[A] = 10^−4^ cm^−1^

Next complex, CuH_−1_L, dominates in pH 7.5 (Fig. 3a). The stoichiometry of this form suggests the loss of 6 protons from the ligand molecule. In these conditions lysyl residue is still protonated, so it can be concluded that besides two Asp and two His residues, two amide nitrogen atoms are deprotonated. However, owing to the location of both imidazole donors in the peptide chain, together with deprotonation of two amide donors, the discussed complex may be characterized by $\left\{ {{\text{N}}_{\text{Im}} ,{\text{ 2N}}_{\text{amide}}^{ - } } \right\}$ binding mode and thereby metal ion is coordinated here only by three nitrogen atoms. The second His residue did not bind metal ion. Spectroscopic results confirm the existence of 3N complex (Table 2). The experimental value of *λ*_max_ = 582 nm is in a good agreement with the theoretical *λ*_max_ = 584 nm calculated for two amides, one imidazole nitrogen and one oxygen from water molecule donor sets (Prenesti et al. 1999). Furthermore, the appearance of two positive bands on CD spectrum at 247 and 323 nm confirms the binding of copper (II) ion by both: imidazole and amide nitrogen atoms, respectively (Table 2). Value of log*K* = 7.18 obtained from potentiometric results also suggest the involvement of the amide nitrogen atom in the metal ion coordination (Table 2) (Kallay et al. 2009; Marciniak et al. 2014).

Around pH 7 CuH_−2_L complex appears in the system. The log*K* value of proton dissociation and formation of this complex is equal to 8.21 and supports binding of the next amide nitrogen and formation of the square planar complex with the $\left\{ {{\text{N}}_{\text{Im}} ,{\text{ 3N}}^{ - }_{\text{amide}} } \right\}$ binding mode (Orfei et al. 2003). The EPR parameters obtained for the system at pH 9 [A_||_ = 199 (cm^−1^) and g_||_ = 2.194, Table 3] confirm coordination of four nitrogen atoms to the metal ion (Peisach and Blumberg 1974). Moreover, the components of g tensor (g_z_ ≫ g_y_ = g_x_ > 2.0023) show the axial symmetry and the characteristic ${\text{A}}_{\text{II}}^{\text{Cu}} \, \gg \,{\text{A}}_{ \bot }^{\text{Cu}}$ splitting pattern suggests the d_x2−y2_ ground state (Godlewska et al. 2013; Pap et al. 2011). The comparison of the theoretical λ_max_ = 523 nm (Prenesti et al. 1999) together with the experimental λ_max_ = 525 nm (Table 2) in absorption spectrum also confirms $\left\{ {{\text{N}}_{\text{Im}} ,{\text{ 3N}}^{ - }_{\text{amide}} } \right\}$ binding mode for this complex. Moreover, positive CT transition at 322 nm (Table 2) shows major involvement of amide nitrogen atoms in the metal ion binding.

Above pH 9 next two protons dissociate from the molecule and CuH_−3_L and CuH_−4_L complexes are formed (Fig. 3a). However, no changes in spectroscopic parameters are observed. The log*K* value of formation of CuH_−3_L complex (10.19, Table 2) suggests proton dissociation from Lys residues. The creation of the CuH_−4_L may be a result of the loss of a proton from the second nitrogen atom in the imidazole ring from the histidine moiety involved in copper ion coordination, what was observed in the case of other His-peptides (Brasuń et al. 2007).

The second somatostatin analog, Ac[H^1,9^D^2,10^]c(SST), has different position of His and Asp moieties in the peptide chain in comparison with the previously described peptide. It creates seven types of complexes with copper(II) ions (Fig. 3b). CuH_3_L coexists in the system with the next one CuH_2_L and with the uncoordinated metal ion so it was not possible to describe it using spectroscopic methods. However, analysis of the potentiometric results may give some suggestion. The value of logβ* = 3.01, where logβ* = log*β*_*CuHnL*_ − log*β*_*HnL*_, suggests the {N_Im_} binding mode (Kapinos et al. 1998; Marciniak et al. 2014). Moreover, the log*K* = 4.45, for the reaction: CuH_3_L → CuH_2_L is comparable to the protonation constants for deprotonation of one carboxylate group from Asp residues (log*K* = 4.53) without binding to the metal ion (Table 4).

**Table 4:** Potentiometric and spectroscopic data for the Cu(II)/Ac[H^1,9^D^2,10^]c(SST) system at 25 °C, I = 0.1 mol/L (KCl)

Ac[H^1,9^D^2,10^]c(SST)	log *β*	log *K*	UV–Vis		CD	
			λ (nm)	ε (M^−1^ cm^−1^)	λ (nm)	Δε (M^−1^ cm^−1^)
HL	10.10 ± 0.01					
H_2_L	17.15 ± 0.02					
H_3_L	23.33 ± 0.01					
H_4_L	27.46 ± 0.02					
H_5_L	30.86 ± 0.02					
log*K*_Lys_		10.10				
log*K*_His_		7.05				
log*K*_His_		6.18				
log*K*_Asp_		4.53				
log*K*_Asp_		3.40				
CuH_3_L	26.34 ± 0.06		–	–	–	–
CuH_2_L	21.89 ± 0.04		–	–	–	–
CuHL	~16.65 ± 0.06		–	–	–	–
CuL	~10.85 ± 0.08		–	–	–	–
CuH_−1_L	3.60 ± 0.02		583	86	588^a^	−0.199
					324^c^	0.713
					256^b^	1.11
CuH_−2_L	−5.39 ± 0.02		556	99	557^a^	−0.435
					282^b,c^	1.69
CuH_−3_L	−15.76 ± 0.02		555	100	557^a^	−0.498
					282^b,c^	1.88
					313sh	1.12
log*K*_CuH3L–CuH2L_		4.45				
log*K*_CuH2L–CuHL_		5.24				
log*K*_CuHL–CuL_		5.80				
log*K*_CuL–CuH–1L_		7.25				
log*K*_CuH–1L–CuH–2L_		8.39				
log*K*_CuH–2L–CuH–3L_		10.37				

The ligand concentration was 8 × 10^−4^ mol/L; 1:1 ligand to metal ratio

*sh* shoulder

^a^d–d transition

^b^N_Im_ → Cu(II) CT

^c^N^−^ → Cu(II) CT

Log*K* = 5.24 for the reaction of CuHL formation suggests proton dissociation from histidine moiety (Table 4). Stoichiometry of the described form indicates the loss of protons from two His residues. Therefore, {2N_Im_} coordination mode is possible here. The value of logβ* = log*K*_CuHL_–log*β*_HL_ = 6.55, which is similar to logβ* for the other complexes with the same coordination mode in systems described in the literature (Brasuń et al. 2007; Kotynia et al. 2014) confirms this type of copper (II) binding.

Similarly to Ac[D^1,9^H^2,10^]c(SST), when CuL complex with neutral charge exists in the system, precipitation was observed. Therefore, determination of spectroscopic parameters for CuL was not possible. Only on the basis of the stoichiometry of the described complex it can be concluded that the first amide nitrogen is involved in the metal ion coordination.

While pH increases, next three complexes: CuH_−1_L, CuH_−2_L; and CuH_−3_L appear in the system (Fig. 3b). Coordination modes for these forms are the same as in case of Ac[D^1,9^H^2,10^]c(SST) analog, what is confirmed by potentiometric and spectroscopic parameters (Tables 4, 5).

**Table 5:** The EPR parameters obtained by simulation of spectra in Cu(II)/Ac[H^1,9^D^2,10^]c(SST) system

Species parameters	CuH_−1_L {N_Im_, 2N^−^}	CuH_−2_L {N_Im_, 3N^−^}
g_z_ (g_||_)	2.224	2.201
g_x_, g_y_ (g_**⊥**_)	2.051	2.042
A_z_^Cu^ (A_||_)^a^	183	201
${\text{A}}_{\text{x}}^{\text{Cu}} ,{\text{ A}}_{\text{y}}^{\text{Cu}} ({\text{A}}_{ \bot } )^{{^{\text{a}} }}$	14	24

^a^[A] = 10^−4^ cm^−1^

**Fig. 4:**
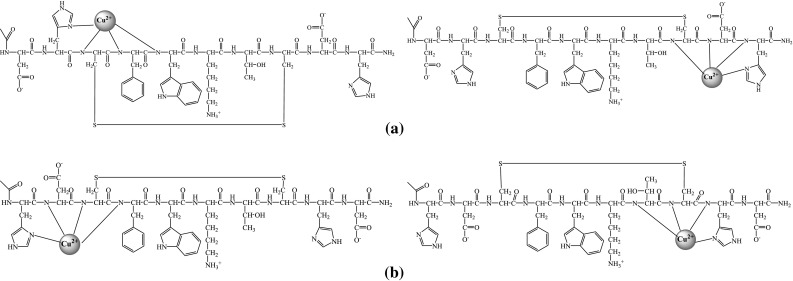
Two possible manners of Cu(II) coordination in CuH_−2_L complexes for: **a** Ac[D^1,9^H^2,10^]c(SST), **b** Ac[H^1,9^D^2,10^]c(SST)

It is difficult to define which histidine moieties are involved in the coordination of copper (II) ion in the particular complexes. In case of 4 N complexes with {N_Im_, 3N^−^} coordination modes two options for each of the peptides are possible (Fig. 4). However, in view of the creation of (6,5,5)-chelate rings in case of copper (II) ion coordination by histidine moieties on C-terminal of peptides chains, this way of binding seems to be energetically more favorable than the second one where (7,5,5)-chelate rings are created (Sovago et al. 2012). Therefore, coordination by His^10^ in Ac[D^1,9^H^2,10^]c(SST) and His^9^ in Ac[H^1,9^D^2,10^]c(SST) is much more probable.

**Fig. 5:**
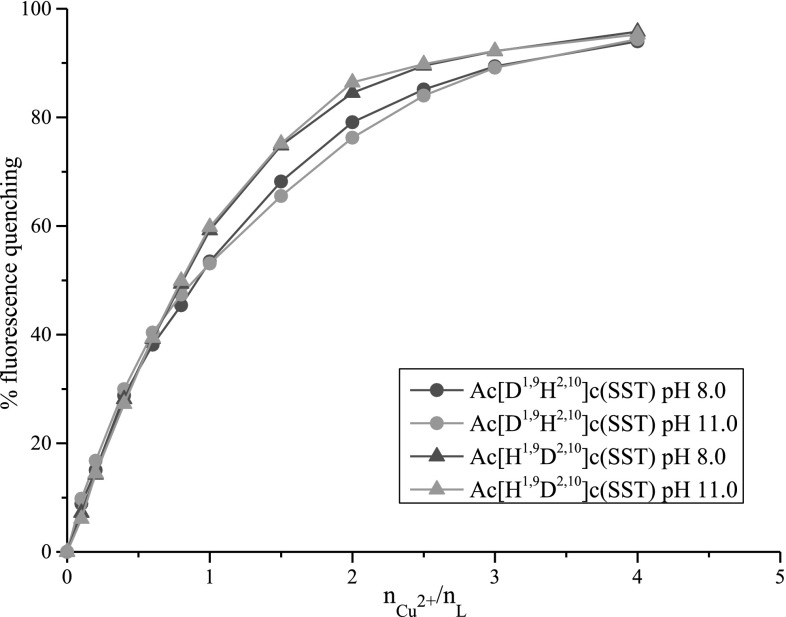
Fluorescence quenching for Ac[D^1,9^H^2,10^]c(SST) and Ac[H^1,9^D^2,10^]c(SST) as a function of increasing Cu^2+^ concentration in pH 8.0 and 11.0

Tryptophan is responsible for the phenomenon of fluorescence in both analyzed peptides (Lakowicz 2006). In the manner of the metal ion binding for CuH_−2_L complexes proposed above no atoms derived from this amino acid residue are involved in the coordination of Cu(II). Therefore, study of the fluorescence quenching in analyzed systems could be an indication that described binding manners are possible. Analysis in pH 8.0 and 11.0 show that in both peptides quenching of fluorescence is very similar (Fig. 5). In the analyzed conditions in both systems the same types of complexes with the same coordination modes are formed. In all cases almost 100 % of fluorescence is quenched. It means that tryptophan residues are located on the surface of the complexes and are not protected from the quencher by the rest of the molecule. It can be concluded that fragments of the peptide chains with tryptophan residues have similar spatial arrangement in both ligands. Therefore, the proposed coordination modes with involvement of histidine moieties on C-terminal are possible.

**Fig. 6:**
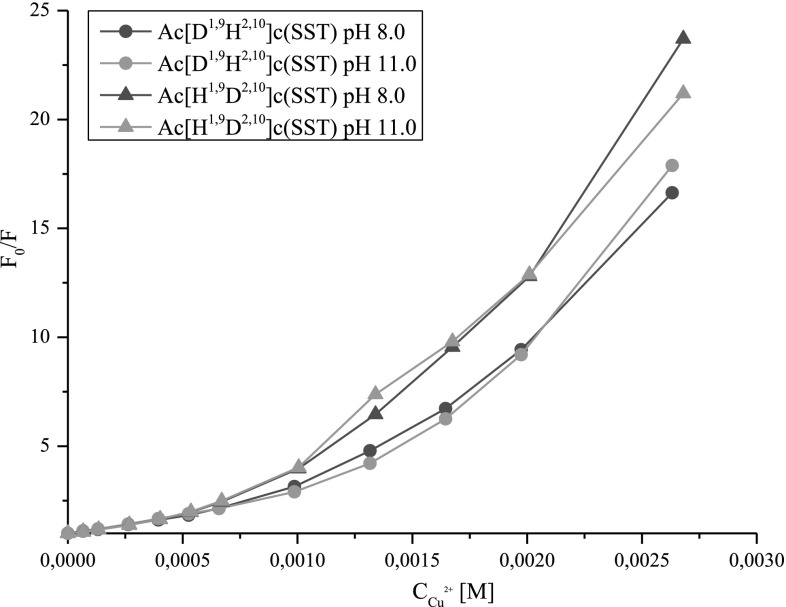
Stern–Volmer plots for copper (II) quenching of fluorescence for Ac[D^1,9^H^2,10^]c(SST) and Ac[H^1,9^D^2,10^]c(SST)

Fluorescence quenching was also analyzed by Stern–Volmer equation (Lakowicz 2006) (Fig. 6). Obtained plots indicate mixed mechanism of quenching: static and dynamic.

**Fig. 7:**
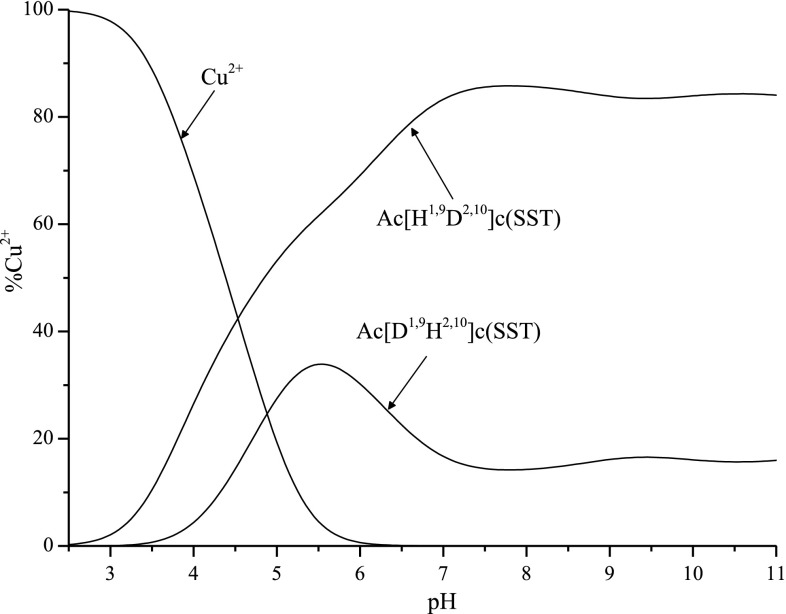
Distribution profile of free and bound fractions of Cu(II) in the presence of Ac[D^1,9^H^2,10^]c(SST) and Ac[H^1,9^D^2,10^]c(SST)

Figure 7 shows the comparison of the efficiency in copper (II) ion binding between the two analyzed peptides. Both ligands have a very similar construction but Ac[H^1,9^D^2,10^]c(SST) coordinates copper (II) ion much better than the second one in the whole analyzed range of pH. It is probably related to the earlier (in lower pH) coordination of amide nitrogen atoms in case of Ac[H^1,9^D^2,10^]c(SST)/Cu^2+^ system (first amide nitrogen coordinates to copper (II) ions in CuL complexes in both systems). Moreover, the ligand with histidine moiety in the first position coordinates the metal ion by amide nitrogen from Cys^8^ in CuH_−1_L complex. This is the first amino acid which is involved in the coordination and it occurres within the cyclic structure. In case of Ac[D^1,9^H^2,10^]c(SST) similar situation is observed later in the CuH_−2_L complex. It can also affect the difference in the effectiveness in copper (II) ion coordination.

Presented results demonstrated that small modifications within the peptide sequence can significantly affect efficiency of the metal ion binding.

## Conclusion

In this paper we have shown that new somatostatin analogs with the characteristic part of the sequence -c(Cys-Phe-Trp-Lys-Thr-Cys)- and with two histidine and two aspartic acid moieties in their structures bind copper(II) ions effectively. Both ligands have very similar construction but Ac[H^1,9^D^2,10^]c(SST) coordinates copper (II) ion much better than the second one in the whole analyzed range of pH. It was shown that small modifications within the peptide sequence can significantly affect efficiency of the metal ion binding. These results may be an introduction to the discovery of new precursors of pharmaceuticals useful in the PRRT.
